# Prioritized Multi-task Motion Coordination of Physically Constrained Quadruped Manipulators

**DOI:** 10.34133/cbsystems.0203

**Published:** 2025-03-19

**Authors:** Aizhen Xie, Xuewen Rong, Guoteng Zhang, Yibin Li, Yong Fan, Zhi Li, Teng Chen

**Affiliations:** ^1^ School of Artificial Intelligence, Shandong University, Jinan 250300, China.; ^2^ School of Control Science and Engineering, Shandong University, Jinan 250061, China.; ^3^ School of Rail Transportation, Shandong Jiaotong University, Jinan 250357, China.; ^4^ Shandong Youbaote Intelligent Robotics Co., Ltd., Jinan 250032, China.

## Abstract

Quadruped manipulators can use legs to mimic legged animals for crossing unstructured environments. They can also use a bionic arm to execute manipulation tasks. The increasing demands for such robots have pushed research progress. However, there remain challenging works in their usage of a high degree of freedom. To solve this redundant problem, we propose a novel motion coordination framework based on multi-task prioritization and null-space projection. The framework can adaptively generate optimal motion for different parts of the robot considering 3 prioritized tasks. The tasks include end-effector trajectory tracking, motion redistribution to meet physical constraints, and manipulability enhancement. The motion is then executed by a whole-body controller incorporating dynamics, inverse kinematics, multiobjective priorities, and force constraints. Experiments both in simulation and on the robot platform validate the advantages and effectiveness of the algorithm. The robot can finish robust and accurate operational space end-effector tracking with errors less than 3 cm.

## Introduction

Legged animals have served as inspiration sources for robots because of their ability to traverse difficult environments. One of their robotic counterparts, which is the quadruped robot, has been developed by many research institutions and companies [1,2]. Despite the impressive progress in their locomotion ability, most quadruped robots lack the ability to manipulate. One solution for this is to mount a manipulator on the robot. The manipulator can have the bionic structure of a human arm to execute different manipulation tasks. There are some examples of such robots, and they are named quadruped manipulators [3–6]. The combination for these robots extends the system’s ability. However, adding an arm results in a very high-degree-of-freedom (high-DOF) system and brings many challenging problems. The modeling and control of the system are difficult. Optimal motion coordination [7] is another key problem for 2 reasons: first, motion distribution between the 2 parts has to deal with the body’s mobility and the arm’s flexibility; second, motion generation of the system involves complex contacts with the environment to meet the needs for stable locomotion, kinematic/dynamic limits, and physical constraints.

Early works on modeling and control of quadruped manipulators include research on BigDog by Murphy et al. [8]. They used a simplified 13-dimensional model and a prioritized version of virtual model control to accomplish dynamic lifting and throwing of heavy blocks. Rehman et al. [9] modeled the quadruped and the arm separately and controlled the robot using their integrated control framework to tackle the influence between the quadruped and the manipulator. In contrast to the 2 aforementioned examples, Bellicoso et al. [10] presented a whole-body controller (WBC) using the robot’s whole kinematics and dynamics model. They showed cooperation and multiple manipulation results in their paper. Risiglione et al. [11] proposed a WBC to coordinate tracking performance and desired compliance for the base and the manipulator. Whole-body controllers can also be found in studies by Chiu et al. [12] and Li et al. [13]. Another example using whole-body dynamics was the one by Hamed et al. [14], which developed a low-level controller based on virtual constraints for optimal trajectory tracking. The biggest advantage of a WBC is that it can deal with the robot’s kinematic and dynamic interaction between the quadruped and the arm internally. The robot can use the hierarchical structure and null-space projection [15] to finish diverse tasks, including end-effector trajectory tracking, force output enhancement, hard constraint fulfillment, singularity avoidance, and obstacle avoidance.

In the context of motion generation, Bellicoso et al. [10] controlled the quadruped body and the manipulator by sending independent commands to the 2 parts. The motions are then generated independently. A similar method can be found in a study by Xie et al. [16]. The biggest disadvantage of such a method is that the motion distribution between the 2 parts is achieved by people. The decision made by people may be wrong for the lack of reaction force feedback or global vision. The manipulator may be driven to stretch out of the workspace or meet singularities. Sleiman et al. [17] used a unified model predictive control framework to plan the motions of a whole robot. The whole-body planning method can also be seen in a study by Mittal et al. [18]. However, using the full DOF of the robot in planning may result in a complex problem and the calculation loop may become long for highly dynamic motion. Another solution is to simplify the kinematic formulation for the robot and make it possible to plan online [19].

Another target of motion coordination is to improve the tracking results. Abe et al. [20] used a prioritized version of virtual model control to track offline motion planning. Rehman et al. [9] presented a controller for the arm using a time-delay estimation scheme to improve tracking error. Xin et al. [21] accomplished the manipulation work by one leg, estimated the contact forces from trajectory errors, and relayed the force feedback to the operator. Ferrolho et al. [22] improved the motion execution procedure by adding a robustness metric to the trajectory optimization work. Ma et al. [23] adjusted both the cost function of model predictive control and the manipulator’s configuration in their learning-based locomotion policies to improve the tracking results. Zimmermann et al. [24] combined feed-forward trajectory planning with a feedback controller to get accurate tracking results. From those examples, we found that the improvement of tracking errors can be done in the process of either planning or tracking. It can also happen in both procedures.

In this paper, we propose a novel coordinated motion distribution and tracking algorithm for quadruped manipulators. We adopt the simplification method by Murphy et al. [8] to establish our system’s kinematics and dynamics model. The quadruped base is modeled as the extended joints of the manipulator. We also take advantage of the WBC and propose a motion execution method based on prioritized task projection. The projection idea is adopted from the “saturation in the null space” algorithm presented by Flacco et al. [25] and extended to quadruped manipulators. It is used in both motion generation and execution to effectively address the redundant system’s limitations. Limitations on different working modes are established. A tracking camera with an integrated environment perception algorithm is used in the system. Motion coordination is to finish 3 prioritized tasks. The first and most important task is trajectory tracking of the end effector. The motion redistribution of the robot is then programmed in the null space to enhance tracking accuracy and meet physical constraints. A compensation velocity is designed as the third task to realize singularity avoidance. The generation results are executed using the proposed WBC. Groups of comparison experiments using the proposed method and the traditional pseudo-inverse method are presented to demonstrate the effectiveness of the framework.

We organize the paper as follows: The kinematic and dynamic formulation for the quadruped manipulator is introduced in Methods. The coordinated motion distribution algorithm is provided in Motion Distribution Algorithm Using Task Prioritization. Motion Execution Using a WBC introduces the tracking controller. The effectiveness and advantages of the framework are shown by simulations and experiments on the quadruped manipulator in Results and Discussion. Conclusion concludes the paper.

## Methods

**Fig. 1.**
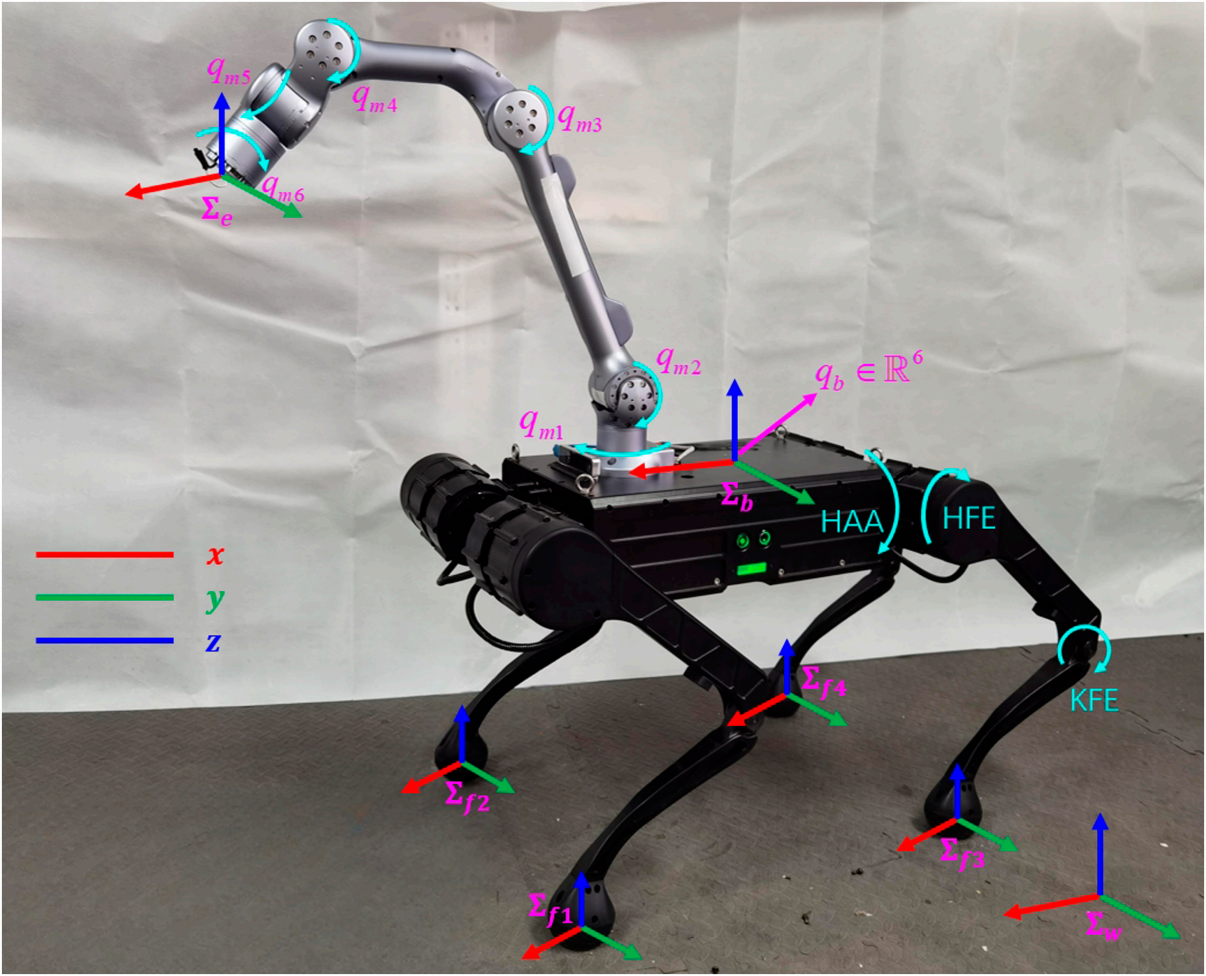
Sketch of a quadruped manipulator and the coordinates used. HAA, hip abduction/adduction; HFE, hip flexion/extension; KFE, knee flexion/extension.

In this section, we establish both the kinematic and dynamic formulation for the quadruped manipulator. Fig. 1 shows a sketch of the robot and the main coordinates used. $\Sigma_{w}$ is used to denote the world reference frame. $\Sigma_{b}$ corresponds to the quadruped base frame with its location on the center of the base. Its *x* axis is aligned with the direction of the robot’s forward motion. $\Sigma_{e}$ is the end-effector frame whose location is on the gripper’s center. Its *z* axis is aligned with the rotation axes of $q_{m6}$. $\Sigma_{fi}$ corresponds to the ith contact frame.

### Kinematic modeling

Our method uses a simplified kinematic model of the robot that abstracts the legs to generate a virtual wrench of the floating base. A 6-DOF base and a 6-DOF arm result in a 12-DOF system with the generalized coordinate defined as

$$q_{\mathrm{bw}}={\left[q_{b}^{T}\;q_{m}^{T}\right]}^{T}\in {\mathbb{R}}^{n}$$ (1)

where $q_{b}\in {\mathbb{R}}^{n_{b}}$ is the generalized floating base coordinate and $q_{m}\in {\mathbb{R}}^{n_{m}}$ is the respective coordinate for the arm, $n=n_{b}+n_{m}$. The simplified forward kinematic formulation is expressed as

$$x_{w}=h\left(q_{\mathrm{bw}}\right)$$ (2)

where $x_{w}\in {\mathbb{R}}^{r}$ is an r×1 spatial vector used to represent the end effector’s pose in the world frame; $h\left(q_{bw}\right)$ denotes the forward kinematics.

The generalized velocity vector for the robot can be expressed as ${\overset{\cdot }{q}}_{\mathrm{bw}}={\left[\begin{array}{cc} {\overset{\cdot }{q}}_{b}^{T} & {\overset{\cdot }{q}}_{m}^{T} \end{array}\right]}^{T}\in {\mathbb{R}}^{n}$. By calculating the differential of Eq. (2), we can get the velocity of the end effector using the following expression:

$${\overset{\cdot }{x}}_{w}=J_{\mathrm{bw}}\left(q_{\mathrm{bw}}\right){\overset{\cdot }{q}}_{\mathrm{bw}}=\left[\begin{array}{cc} J_{b}\left(q_{\mathrm{bw}}\right) & J_{m}\left(q_{\mathrm{bw}}\right)\; \end{array}\right]\left[\begin{array}{c} {\overset{\cdot }{q}}_{b}\\ {\overset{\cdot }{q}}_{m} \end{array}\right]$$ (3)

where $J_{b}\in {\mathbb{R}}^{n_{b}\;\times \;n_{b}}$, $J_{m}\in {\mathbb{R}}^{n_{m}\;\times \;n_{m}}$, and $J_{\mathrm{bw}}\in {\mathbb{R}}^{n_{b}\;\times \;n}$ are the Jacobian matrices of the floating base, the manipulator, and the robot, respectively. The updating procedure for the Jacobian matrix can be found in a paper by Carpentier and Mansard [26].

Given the end-effector desired velocity ${\overset{\cdot }{x}}_{w}$, the solution of the inverse kinematics can resort to an optimization technique and be expressed as

$${\overset{\cdot }{q}}_{\mathrm{bw}}=J_{\mathrm{bw}}^{†}{\overset{\cdot }{x}}_{w}+\left(I-J_{\mathrm{bw}}^{†}J_{\mathrm{bw}}\right){\overset{\cdot }{q}}_{\mathrm{bw}}^{\text{null}}$$ (4)

where $J_{\mathrm{bw}}^{†}$ is the Moore–Penrose inverse of $J_{\mathrm{bw}}$ and ${\overset{\cdot }{q}}_{\mathrm{bw}}^{\text{null}}$ is the velocity command in the Jacobian null space. The expression $\left(I-J_{\mathrm{bw}}^{†}J_{\mathrm{bw}}\right)$ represents the orthogonal projection matrix in the null space of $J_{\mathrm{bw}}$.

### Dynamic modeling

Concerning the dynamics modulation, we use the whole robot’s DOF to form the full equation of motion. The generalized coordinate is defined as

$$q={\left[\begin{array}{ccc} q_{b}^{T} & q_{l}^{T} & q_{m}^{T} \end{array}\right]}^{T}\in {\mathbb{R}}^{n+n_{l}}$$ (5)

where $q_{l}\in {\mathbb{R}}^{n_{l}}$ is the generalized coordinate for the legs. The dynamics equation can be expressed as

$$M\left(q\right)\ddot{q}+H\left(q,\;\overset{\cdot }{q}\right)=S^{T}\tau +J_{\mathrm{st}}^{T}F_{\mathrm{grf}}$$ (6)

where $M\in {\mathbb{R}}^{\left(n+n_{l}\right)\;\times \;\left(n+n_{l}\right)}$ denotes the inertia matrix; $H\in {\mathbb{R}}^{n+n_{l}}$ represents the centripetal, Coriolis, and gravitational forces; S is the selection matrix for choosing which DOFs are actuated; $\tau ={\left[\begin{array}{ccc} 0_{b} & \tau_{l}^{T} & \tau_{m}^{T} \end{array}\right]}^{T}\in {\mathbb{R}}^{n+n_{l}}$ includes the unactuated and actuated parts, $\tau_{l}\in {\mathbb{R}}^{n_{l}}$ are the actuated joint torques of the legs, and $\tau_{m}\in {\mathbb{R}}^{n_{m}}$ are the actuated joint torques of the manipulator; $F_{\mathrm{grf}}\in {\mathbb{R}}^{3\;\times \;n_{\mathrm{st}}}$ is the vector of ground reaction forces expressed in the contact frame; $n_{\mathrm{st}}$ is the number of stance legs; and $J_{st}$ is the contact Jacobian.

### Motion distribution and execution framework

**Fig. 2.**
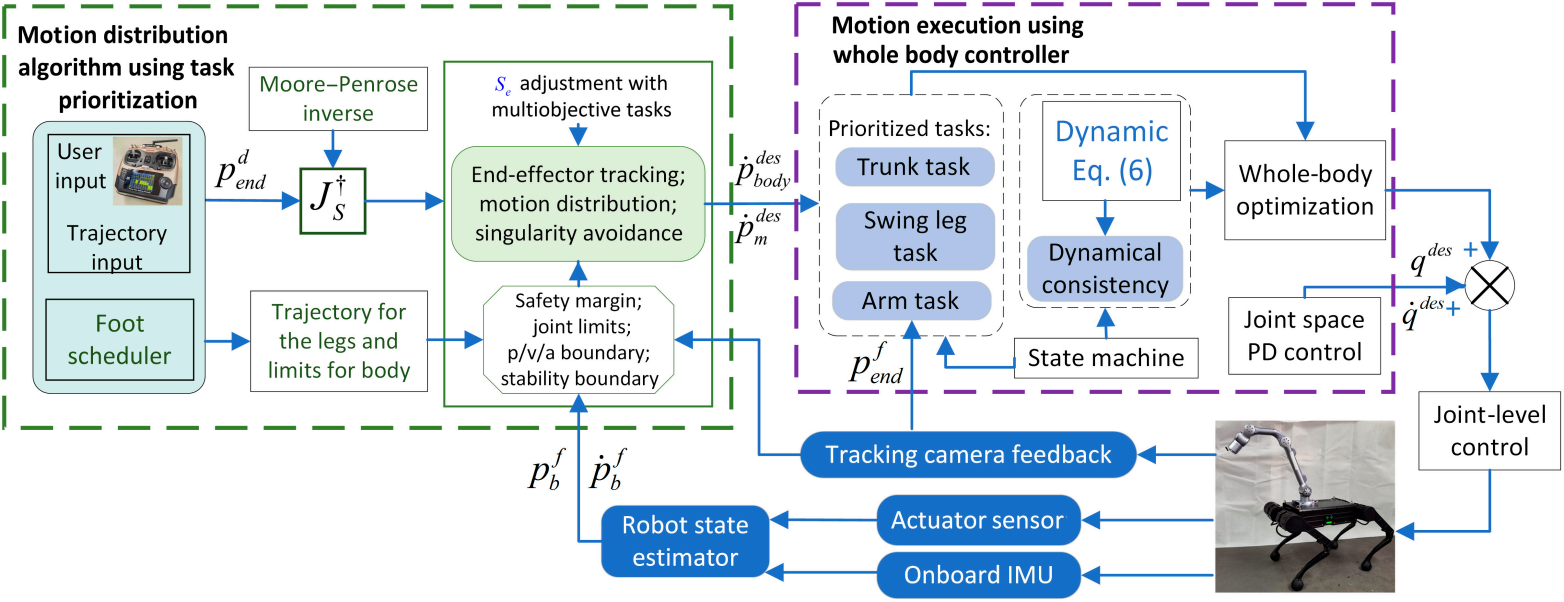
Block diagram of the motion distribution and control framework. PD, proportional–derivative; IMU, inertial measurement unit.

Fig. 2 depicts the main components of our motion distribution and execution framework. A high-level velocity command is given by the remote controller or a predefined trajectory. The motion distribution part uses the command to generates the motion for the robot and provides it to the WBC. The motion distribution between the body and the manipulator is carried out adaptively by the algorithm. The velocity manipulability is enhanced at the same time. Details of the motion distribution are presented in Motion Distribution Algorithm Using Task Prioritization.

The goal of the WBC is to accurately track the trajectory results while interacting compliantly with the environment. The state machine is used to switch between different working modes. The state estimator uses the onboard inertial measurement unit and the proprioceptive sensors to estimate the robot’s state information. The WBC optimizes the forces and the joint accelerations that satisfy all robot constraints (torque limits, kinematic limits, and friction cone). The outputs are mapped into desired feed-forward torque commands. The commands are combined with a joint impedance controller to get the final torque orders and then sent to the low-level joint controllers.

### Motion distribution algorithm using task prioritization

In this section, we present the adaptive motion distribution method using an inverse kinematics solution and task projection in null space. Given the task of end-effector trajectory tracking, we divide it into 3 sub-tasks and use a hierarchical structure for task execution. We also add a feedback term for the desired end-effector pose in Cartesian space to improve tracking accuracy and make sure that the error converges to zero. The approach can be expressed by a modified solution of the inverse kinematics in the following equation:

$${\overset{\cdot }{q}}_{\mathrm{bw}}=J_{S}^{†}\left({\overset{\cdot }{x}}_{d}+k_{x}\left(x_{d}-x\right)\right)+\left(I-J_{S}^{†}J_{S}\right){\overset{\cdot }{q}}_{\mathrm{cmd}}^{\text{null}}+{\overset{\cdot }{q}}_{\mathrm{cmd}}^{\mathrm{sv}}$$ (7)

where $J_{S}$ is the modified Jacobian matrix; $x\in {\mathbb{R}}^{r}$ and $x_{d}\in {\mathbb{R}}^{r}$ denote the actual and desired values, respectively; $k_{x}\in {\mathbb{R}}^{r\;\times \;r}$ is a diagonal positive-definite matrix; ${\overset{\cdot }{q}}_{\mathrm{cmd}}^{\text{null}}$ represents the velocity commands for the second sub-task, which is motion redistribution; and ${\overset{\cdot }{q}}_{\mathrm{cmd}}^{\mathrm{sv}}$ represents the velocity commands for the singularity avoidance task. The details of ${\overset{\cdot }{q}}_{\mathrm{cmd}}^{\text{null}}$ are explained in the “Motion distribution by projection matrix adjustment” section. The details of ${\overset{\cdot }{q}}_{\mathrm{cmd}}^{\mathrm{sv}}$ are explained in the “Singularity avoidance task definition” section.

In this paper, we use the Moore–Penrose inverse in Eq. (7) since the inverse of the nonsquare Jacobian $J_{S}$ does not exist in the redundant case. During the motion distribution procedure, we do not consider the dynamics of the system yet. The dynamically consistent pseudo-inverse is used in our tracking controller as depicted by Xie et al. [16].

#### Motion distribution by projection matrix adjustment

Motion distribution between the quadruped base and the manipulation arm is necessary because the arm has more motion accuracy compared with the base. However, its workspace is limited. The base has larger working ranges but less motion accuracy due to some reasons. The motion of the base is generated by 4 legs. Different walking gaits of the legs would have different motion accuracies. The slippage of the contact legs would result in instability of the base. The state estimation of the base also has impact on the motion accuracy. As a result, we define the projection matrix $J_{S}$ by adding a selection matrix $S_{e}$ to specify the enabled joints:

$$J_{S}=J_{\mathrm{bw}}S_{e}$$ (8)

where $S_{e}$ is an n×n diagonal matrix. The values for its diagonal elements are set to 1 or 0 to activate or deactivate the corresponding DOF, respectively. In detail, $S_{e}$ can be divided into 2 main parts expressed by

$$S_{e}=\left[\begin{array}{cc} S_{\mathrm{be}} & 0\\ 0 & S_{me} \end{array}\right]$$ (9)

The adaptive regulation of $S_{e}$ is realized through a forward searching procedure. The beginning is setting the elements of $S_{me}$ to 1. The elements of $S_{\mathrm{be}}$ are set to 0. Then, the searching repeats calculating the inverse kinematics and looping over every DOF of the manipulator to make sure that they satisfy all constraints. When the DOF exceeds the constraints, the corresponding element of $S_{me}$ will be set to 0. The deficiency of the DOF will be conducted to other joints by defining the value of ${\overset{\cdot }{q}}_{\mathrm{cmd}}^{\text{null}}$ using the limitation bounds. The corresponding jth element of ${\overset{\cdot }{q}}_{\mathrm{cmd}}^{\text{null}}$ is defined by

$${\overset{\cdot }{q}}_{\mathrm{cmd},j}^{\text{null}}=\left\{\begin{array}{cc} {\overset{\cdot }{Q}}_{\text{max},j}, & \text{if}\;{\overset{\cdot }{q}}_{j,\mathrm{cmd}}>{\overset{\cdot }{Q}}_{\text{max},j}\\ {\overset{\cdot }{Q}}_{\text{min},j}, & \text{if}\;{\overset{\cdot }{q}}_{j,\mathrm{cmd}}<{\overset{\cdot }{Q}}_{\text{min},j}\\ 0, & \text{otherwise} \end{array}\right.$$ (10)

where ${\overset{\cdot }{Q}}_{\text{max},j}$ and ${\overset{\cdot }{Q}}_{\text{min},j}$ are the hard constraints for the robot’s DOF and are explained in detail in the next subsection.

During the searching, the rank of $J_{S}$ is calculated to decide the value of $S_{\mathrm{be}}$. When the rank of $J_{S}$ is lower than the dimension of the desired end-effector pose, the motion of the quadruped base is activated and the elements of $S_{\mathrm{be}}$ are set to 1. The looping procedure over the DOF of the manipulator is then extended to include the DOF of the base. The looping is repeated until no DOF exceeds the constraints. The motion distribution procedure for each control loop is concluded in Algorithm 1.


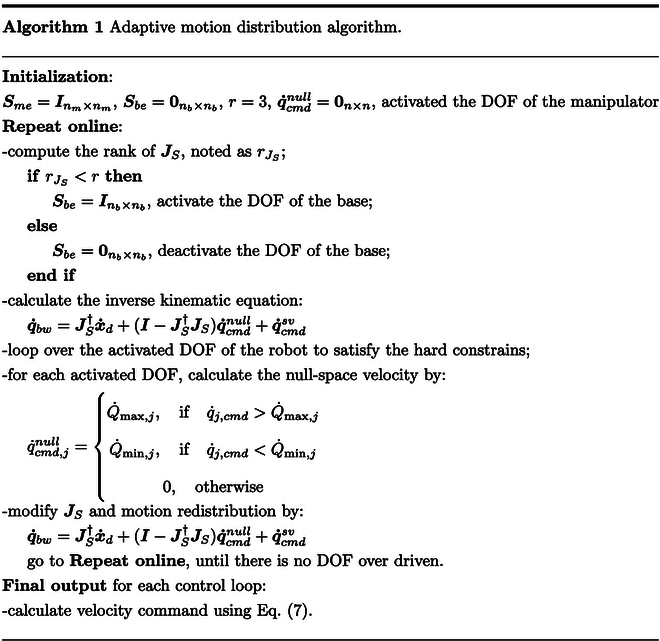


#### Hard constraints for the robot’s DOF

The robot works in the presence of hard constraints for its DOF. The results of the motion distribution are velocity commands. Therefore, we establish the limits on the base and the manipulator in a unified formulation at the velocity level. The joint limits for the manipulator are shaped using the position, velocity, and acceleration bounds, while the limits for the base are obtained concerning the same 3 bounds together with additional locomotion constraints and safety margins. The velocity limits ${\overset{\cdot }{q}}_{bw}$ on the robot’s simplified kinematic model at the current configuration $q_{bw}$ can be shaped as

$${\overset{\cdot }{Q}}_{bw,\text{min}}\left(q_{\mathrm{bw}}\right)\leq {\overset{\cdot }{q}}_{\mathrm{bw}}\leq {\overset{\cdot }{Q}}_{\mathrm{bw},\text{max}}\left(q_{\mathrm{bw}}\right)$$ (11)

where${\overset{\cdot }{Q}}_{\mathrm{bw},\text{min}}$ represents the lower velocity bounds and ${\overset{\cdot }{Q}}_{\mathrm{bw},\text{max}}$ represents the upper limits. Both of them are functions of the current configurations. To the ith DOF, the limits are defined as

$$\begin{array}{c} {\overset{\cdot }{Q}}_{\mathrm{bw},\text{min},i}\left(q_{h,i}\right)=\text{max}\left\{\frac{Q_{\mathrm{bw},\text{min},i}-q_{h,i}}{T},-V_{\mathrm{bw},\text{max},i},{\overset{\cdot }{Q}}_{g,\text{min}}\right.,\\ \;-\sqrt{2A_{\mathrm{bw},\text{max},i}\left(q_{h,i}-Q_{\mathrm{bw},\text{min},i}\right)}{\overset{\cdot }{Q}}_{\text{sm},\text{min}} \end{array}$$ (12)

$$\begin{array}{c} {\overset{\cdot }{Q}}_{\mathrm{bw},\text{max},i}\left(q_{h,i}\right)=\text{min}\left\{\frac{Q_{\mathrm{bw},\text{max},i}-q_{h,i}}{T},V_{\mathrm{bw},\text{max},i},{\overset{\cdot }{Q}}_{g,\text{max}}\right.\\ \;\left.\sqrt{2A_{\mathrm{bw},\text{max},i}\left(Q_{\mathrm{bw},\text{max},i}-q_{h,i}\right)},{\overset{\cdot }{Q}}_{\text{sm},\text{max}}\right\} \end{array}$$ (13)

where $Q_{\mathrm{bw},\text{max}}/Q_{\mathrm{bw},\text{min}}$represent the minimum and maximum position bounds on every DOF that are used to determine the term $\left(Q_{\mathrm{bw},\text{max},i}-q_{h,i}\right)/T$, respectively; $V_{\mathrm{bw},\text{max}}/V_{\mathrm{bw},\text{min}}$ are used for velocity bounds and $A_{\mathrm{bw},\text{max}}/A_{\mathrm{bw},\text{min}}$ correspond to the acceleration bounds; i=1,…,n denotes the joint number; *T* is the duration of sampling time; and $q_{h,i}$ is the robot’s ith joint position at current time $t_{h}$. The detailed calculation for each component in Eqs. (12) and (13) can be found in a paper by Flacco et al. [25]. ${\overset{\cdot }{Q}}_{\text{sm},\text{min}}$ and ${\overset{\cdot }{Q}}_{\text{sm},\text{max}}$ are the limits coursed by locomotion stable margin and safety margins [27]; ${\overset{\cdot }{Q}}_{g,\text{min}}$ and ${\overset{\cdot }{Q}}_{g,\text{max}}$ are the limits coursed by walking gaits. In practical applications, we can add more constraints, such as self-collisions, obstacle avoidance, and terrain adaptation.

#### Singularity avoidance task definition

The distribution algorithm performs the desired end-effector motion in the way of maximizing the motion of the manipulator. However, it may sometimes drive the manipulator to get close to the workspace-boundary singularities. We solve this singularity avoidance problem by checking the singular value and adding the avoidance task in null space. Firstly, at the beginning of each control loop, the singular value of the Jacobian matrix will be calculated to check if the manipulator meets its singularities. If it is true, the base will be activated. Secondly, a tertiary task using the gradient of the manipulability in the null space of the robot is added to the framework.

The manipulability mentioned above is the velocity manipulability ellipsoid [28] defined as

$$H\left(q_{m}\right)=\sqrt{\text{det}\left(J_{m}J_{m}^{T}\right)}$$ (14)

It is used to measure the dexterity of the manipulator from singularities. Its gradient expression is

$$\nabla_{q_{m}}H=\frac{\partial H}{\partial q_{m}}=\left(\frac{\partial H}{\partial q_{1}},\cdots ,\frac{\partial H}{\partial q_{i}},\cdots ,\frac{\partial H}{\partial q_{m}}\right)$$ (15)

for i=1,…,m, $\partial H/\partial q_{i}$ is the partial derivative of *H* to $q_{i}$. Its detailed calculation method can be found in a paper by Jia et al. [29]. We then use it together with a damping term to form the velocity compensation vector defined as

$${\overset{\cdot }{q}}_{S}=k_{N}\left[\begin{array}{c} 0_{n_{b}\times 1}\\ {\left(\nabla_{q_{m}}H\right)}^{T} \end{array}\right]-k_{D}{\overset{\cdot }{q}}_{\mathrm{bw}}$$ (16)

where $k_{N}$ and $k_{D}$ are positive constants. $k_{D}{\overset{\cdot }{q}}_{bw}$ is used to make the system more stable. The compensation vector is used in the robot’s null space. The definition of the null space is $P_{N}=I-J_{m}^{†}J_{m}$. The tertiary object in the null space is defined as

$${\overset{\cdot }{q}}_{\mathrm{cmd}}^{\mathrm{sv}}=\psi \left[I-{\left(\left(I-S_{e}\right)P_{N}\right)}^{†}\right]P_{N}{\overset{\cdot }{q}}_{S}$$ (17)

where ψ is a scaling factor with a range of $\left[0,1\right]$. It is used to downscale the joint’s motion when the remaining redundancy cannot finish the third task. The velocity commands are used in Eq. (7) to represent the singularity avoidance task.

To make the system more reliable, we add another similar scaling factor φ to the desired value of ${\overset{\cdot }{x}}_{d}$ in Eq. (7). It is used on the condition that using all DOFs of the robot cannot finish the desired end-effector trajectory. The final formulation of the velocity command is expressed as

$${\overset{\cdot }{q}}_{\mathrm{bw}}=J_{S}^{†}\left(\varphi {\overset{\cdot }{x}}_{d}+k_{x}\left(x_{d}-x\right)\right)+\left(I-J_{S}^{†}J_{S}\right){\overset{\cdot }{q}}_{\mathrm{cmd}}^{\text{null}}+{\overset{\cdot }{q}}_{\mathrm{cmd}}^{\mathrm{sv}}$$ (18)

The regulation for φ and ψ depends on the limits for the robot’s DOF and the remaining ability to execute all tasks. Details of the regulation can be found in a paper by Xing et al. [30].

### Motion Execution Using a WBC

The motion generation part gets the desired moving speed of the manipulator and the base, which is expressed in the variable ${\overset{\cdot }{q}}_{\mathrm{bw}}$. The results are then sent forward to the WBC for execution. The speed commands ${\overset{\cdot }{q}}_{m}$ for the manipulator are integrated to get the desired position $q_{m}$ for the joints. The speed commands ${\overset{\cdot }{q}}_{b}$ for the base are used with the gait selection from the remote controller to program the motions for the legs. The desired commands for the manipulator, the base, and the legs are then used to compute the torque commands for the robot’s actuated joints under the multiple constraints [31].

#### Motion generation of the legs

The remote controller sends both desired commands for the end-effector and gait selection. A gait scheduler introduced by Chen et al. [32] specifies the time sequences of the stance and swing leg in one cycle. The desired footstep location is expressed by

$$p_{\mathrm{fsw}}=k\sqrt{\frac{h}{g}}\left({\overset{\cdot }{q}}_{b}^{\text{act}}-{\overset{\cdot }{q}}_{b}\right)+\frac{1}{2}{\overset{\cdot }{q}}_{b}\Delta t$$ (19)

where *k* is a regulation gain, ${\overset{\cdot }{q}}_{b}^{\text{act}}$ is the base’s velocity feedback, *h* is the current height of the hip with respect to the ground, and Δt is the control time step. The swing leg trajectory is then defined using the step height, current position and velocity, and the step location to form a cubic polynomial [33].

#### Prioritized tasks and null-space projection

The desired motion of the robot together with the constraints of the system are separated into several tasks, and each task has a priority definition. The highest priority is defined on the contact constraint. The following tasks include the base orientation task, base position task, swing leg trajectory tracking task, and arm joint position task. As already stated by Sentis et al. [15] and Hutter et al. [34], the null-space projection technique can be used to execute prioritized tasks with the projector $N_{i}$ defined as $N=I-J^{†}J$. Therefore, combining with Eq. (6), we calculate the desired robot’s joint commands in a recursive way:

δqi=δqi−1+Ji∣prev†ei−Jiδqi−1q·i=q·i−1+Ji∣pre†x·id−Jiq·i−1q¨i=q¨i−1+Ji∣predyn¯x¨id−J·iq·−Jiq¨i−1 (20)

with

$$\begin{array}{c} \begin{array}{c} J_{i|\text{prev}}=J_{i}N_{\text{prev}\left(k\right)}\\ N_{\text{prev}\left(k\right)}=\prod_{s=2}^{k-1}N_{s|\text{prev}\left(s\right)}\\ N_{s|\text{prev}\left(s\right)}=I-{\left(J_{s}N_{\text{prev}\left(s\right)}\right)}^{†}\left(J_{s}N_{\text{prev}\left(s\right)}\right) \end{array} \end{array}$$ (21)

where $\bar{J_{i|\text{pre}}^{\text{dyn}}}$ is the dynamic consistent inverse that is used to recursively calculate acceleration commands for the prioritized tasks.

#### Whole-body optimization

The results of the above equation are used in a quadratic programming problem [35] to calculate the generalized acceleration and ground reaction forces defined as

$$\begin{array}{c} \;\underset{\delta_{f},\delta_{a}}{\text{min}}\;\delta_{f}^{⊤}W_{f}\delta_{f}+\delta_{a}^{⊤}W_{a}\delta_{a}\\ \begin{array}{c} s.t.\;S_{f}\left(M\left(q\right)\ddot{q}+h\left(q,\;\overset{\cdot }{q}\right)\right)=S_{f}J_{c}^{⊤}\delta_{f}\\ {\mathrm{U\delta}}_{f}\geq 0\\ \ddot{q}={\ddot{q}}^{\mathrm{cmd}}+\left[\begin{array}{c} \delta_{a}\\ 0_{\mathrm{nj}} \end{array}\right] \end{array} \end{array}$$ (22)

where $\delta_{f}$ and $\delta_{a}$ are regulation variables corresponding to the reaction forces and accelerations, $W_{\;f}$ and $W_{\;a}$ are weighting matrices, and U is the pyramid friction cone constraint matrix. The final $\ddot{q}$ and ground reaction forces of the robot are obtained from Eq. (22). They are used in Eq. (6) to get the joints’ feed-forward torque commands $\tau_{\mathrm{ff}}$. A proportional–derivative joint-position controller is also used for better tracking of leg motion. The final commands for all DOFs of the robot are sent to the low-level joint controller through a Controller Area Network bus interface.

## Results and Discussion

The proposed motion distribution and control framework is verified on a quadruped manipulator as is depicted in Fig. 1. For comparison, we also carried out a traditional motion generation test, which employed the pseudo-inverse of the robot’s Jacobian together with manipulability enhancement. We verify the framework by 2 experimental groups. The first group is used to show the necessity of singularity avoidance. The second one is trajectory tracking validation. We also present an implementation analysis about software and hardware for real-time usage on the robot platform.

### Experimental setup and implementations

The experimental setup can also be seen in Fig. 1. The robot used consists of a 40-kg quadruped and a 5-kg mounted arm. All of the joints for the legs and the arm are torque controllable. Both the quadruped and the arm have real-time motor feedback data including position, velocity, and torque. The T265 camera mounted on the manipulator is a tracking camera with an integrated algorithm to measure the real-time position of it in $\Sigma_{w}$. The data are then used in the feedback of the end effector. The robot state estimator uses an onboard inertial measurement unit and motor sensors to calculate the state information of the base.

**Fig. 3.**
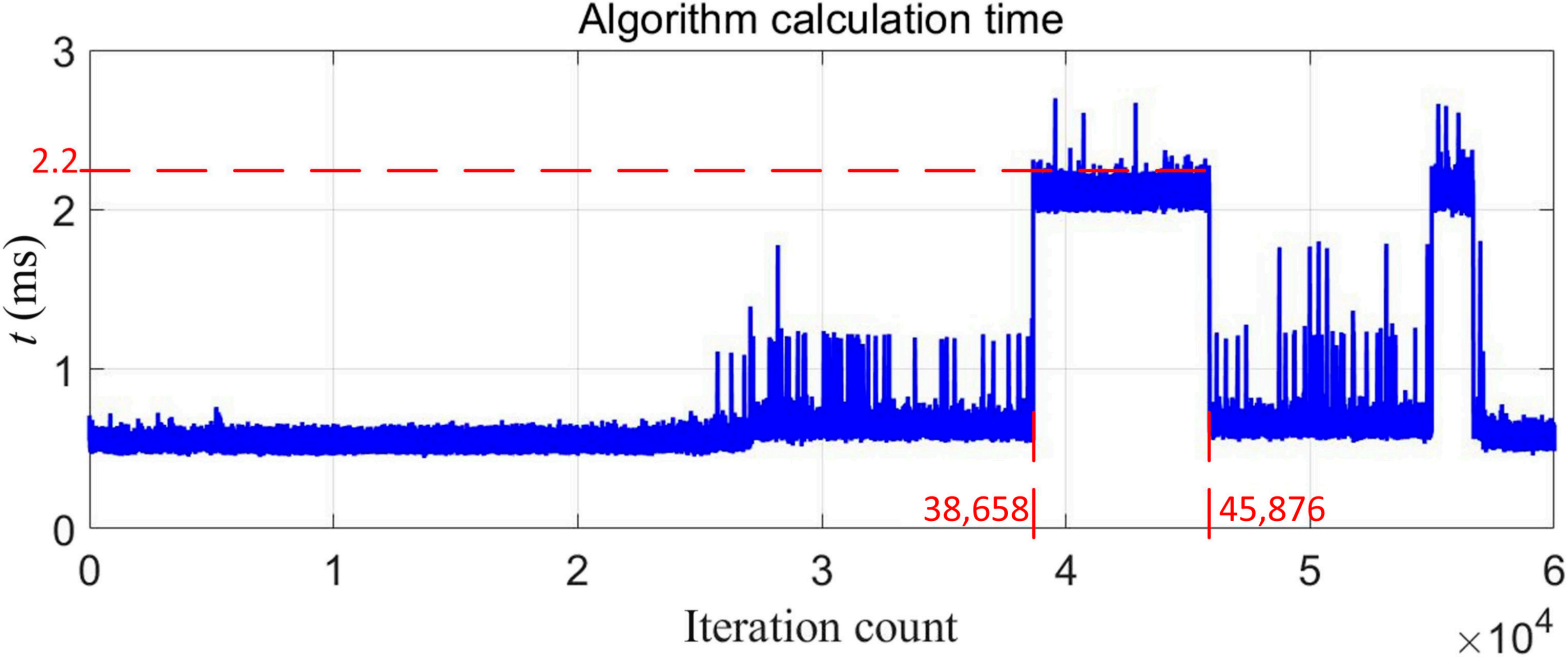
Calculation time of the proposed distribution and execution method.

The robot has 2 powerful consumer onboard personal computers using Intel x5-Z8500 processors with Linux. We choose this setting to reduce the computational and communication burden. We also adopt 2 methods to solve high-dimensional computation problems. Firstly, we used the Eigen library [36] for computations for the matrix and modified the open-source library written by the MIT biomimetic lab [37] to make it compatible with our framework. Secondly, the multithreading approach is employed to decrease the computation time. The recorded time of the distribution and execution controller is depicted in Fig. 3. We can see that the longest time is less than 3 ms. As a result, the controller runs at 300 Hz on the robot platform.

### Necessity validations for singularity avoidance in simulation

Singularity avoidance is indispensable in motion generation. Without that, the manipulator may face infeasible joint commands, resulting in control instabilities. By considering singularity avoidance, the base will be activated and the tracking accuracy will be decreased. However, a trade-off is necessary. The variable $\sigma_{m,\text{min}}$ is used to deal with this trade-off, and we tried different values for testing in the experiments of singularity avoidance.

The presented method without and with singularity experiments were conducted in simulation using the software Webots. We tested the end-effector trajectory using a circle of 0.3-m radius that is out of the manipulator’s workspace. The corresponding motion distribution parameters are depicted in Table 1.

**Table 1.** Parameters used in motion distribution

Parameters	$k_{x}$	$k_{N}$	$k_{D}$	$\sigma_{m,\text{min}}$
Values	$10I_{3\times 3}$	5	0.5	0.1

**Fig. 4.**
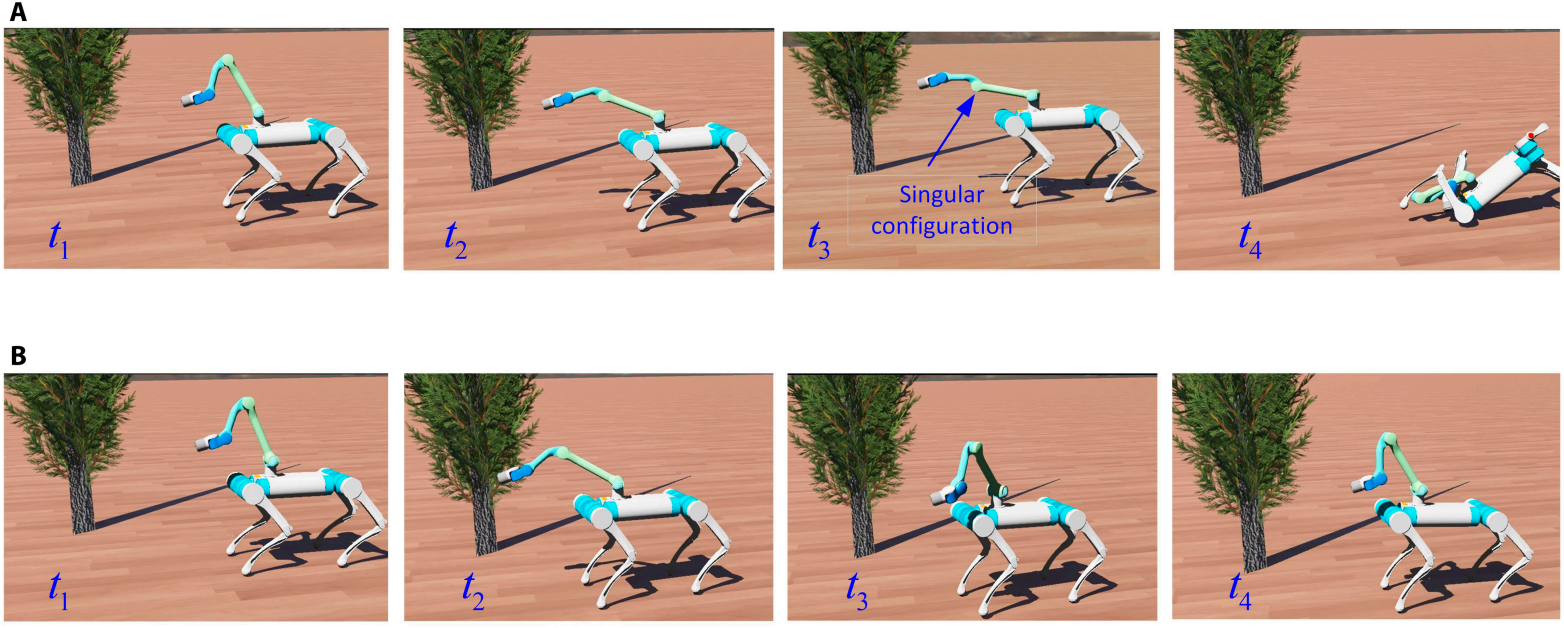
Snapshots of the test in Webots. (A) Without singularity avoidance. (B) With singularity avoidance.

**Fig. 5.**
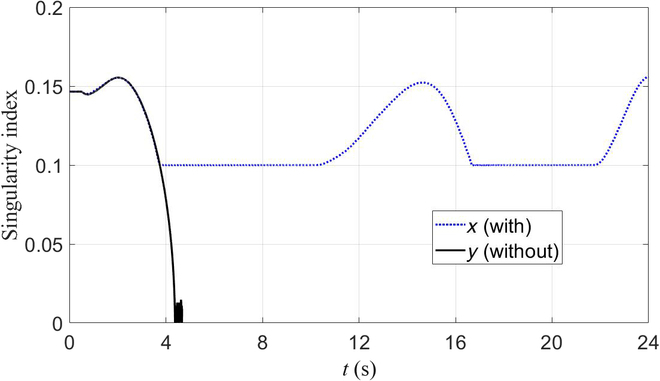
The value of $\sigma_{m}$ during the test.

Firstly, we tested the method without singularity avoidance. At time *t* = 4.2 s, the manipulator’s second, third, and fourth joints are in the form of full extension. Such a situation is one of the system’s kinematically singular configurations with the Jacobian’s singular value approaching zero. The robot accounted unpredictable movements of the manipulator. It then fell down as depicted in Fig. 4A. We then tested the presented method, and the snapshots are depicted in Fig. 4B. The base was activated to assist the manipulator to finish the whole trajectory. Fig. 5 shows the value of $\sigma_{m}$ during the test. It was adopted as the singularity index. We can see that its value reaches zero in the test without singularity avoidance. In contrast, the test with singularity avoidance resulted in its regulation during the procedure. When the value of $\sigma_{m}$ reached its lower bound, the robot activated the base and regulated the arm’s motion to move away from singular configurations. As a result, the value of $\sigma_{m}$ increased. We do not discuss tracking results in this section because it will be presented in detail in the next validation group. The analysis includes experiments on the robot platform and data results.

### Experiments on trajectory tracking

**Fig. 6.**
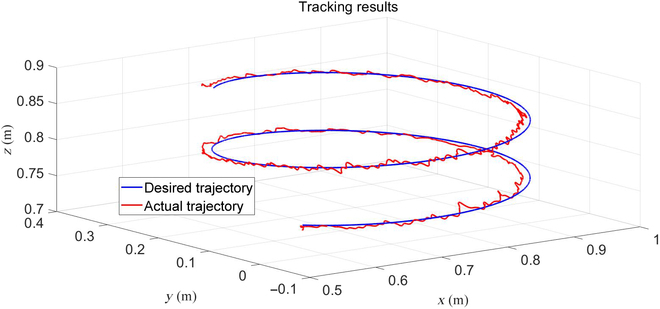
End-effector trajectories using trot gait.

**Fig. 7.**
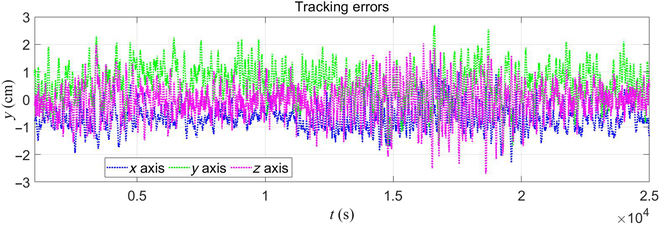
End-effector’s tracking errors using trot gait.

**Fig. 8.**
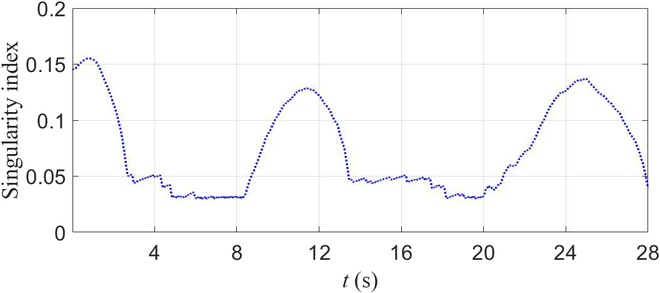
The value of $\sigma_{m}$ during the experiment.

**Fig. 9.**
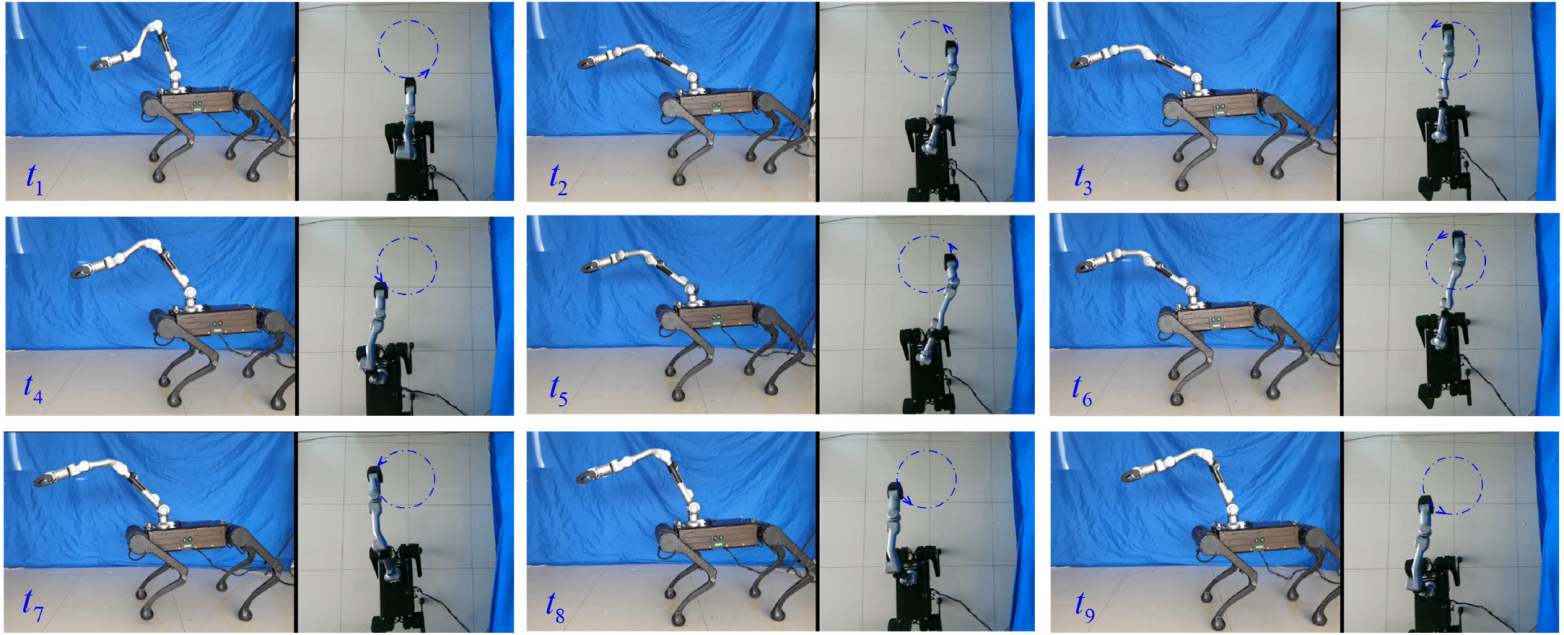
Snapshots of the experiment from a front view (left) and a top view (right).

We carried out 2 experiments in this section to verify the tracking ability of the proposed framework. The first one used a predefined end-effector trajectory expressed as $\begin{array}{c} \begin{array}{c} x_{d}\left(t\right)=x_{0}+{\left[\begin{array}{c} -0.31\text{cos}\left(\pi /20t\right)-0.31\text{sin}\left(\pi /20t\right)\text{·}0.002t \end{array}\right]}^{T} \end{array} \end{array}$. It is a circle on the *xy* plane with a radius of 0.31 m. It was only carried out using the proposed framework on the robot platform with a trot gait, and the results are shown in Figs. 6 to 8. The traditional method that uses the pseudo-inverse of the Jacobian was not done because when we did that in the simulation, the robot met its singularities, which resulted in unpredictable movement, and the robot fell down. When we used the stand gait in simulation, the trajectory could not be finished due to the workspace limitations. The trot gait can finish the task because limits on the *x* axis and *y* axis are almost infinite. Fig. 6 shows the end-effector trajectories, and Fig. 7 shows the errors. Fig. 8 shows the value of $\sigma_{m}$ during the test. With the proposed framework, the robot can finish the trajectory successfully and have maximum errors of 2.2, 2.9, and 2.5 cm in the *x* axis, *y* axis, and *z* axis, respectively. The snapshots of the experiment are shown in Fig. 9 recorded by 2 cameras from a front view and a top view.

**Fig. 10.**
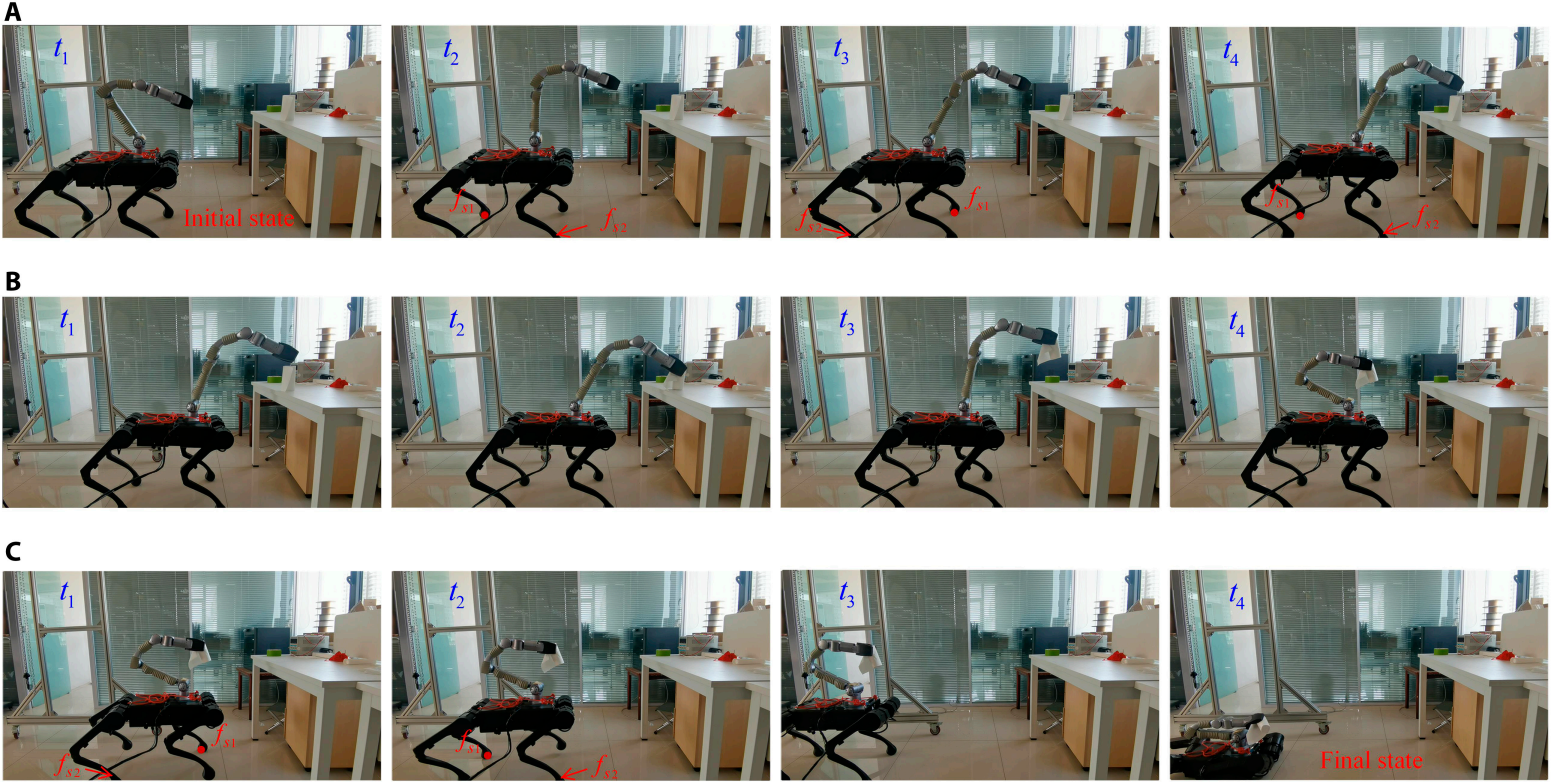
Snapshots of the picking-up-an-object experiment. (A) End-effector tracking to approach the target. (B) Grasping object in balance stand. (C) Locomotion to the next place.

**Fig. 11.**
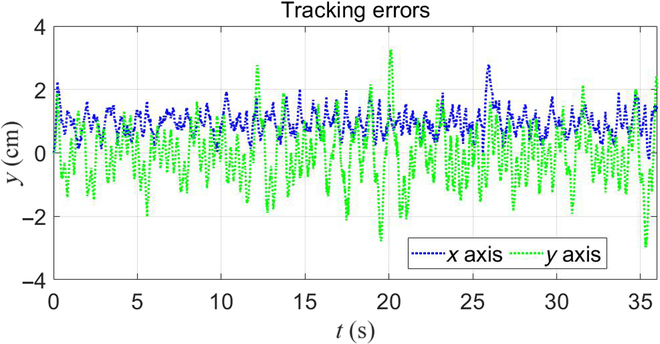
End-effector’s tracking errors during the first stage of the experiment.

Instead of using a predefined trajectory, the second experiment used the remote controller to set end-effector commands and use different working modes to test the robot’s locomotion and manipulation ability. The robot was ordered to pick an object on a desk. The task was separated into 3 stages. The first stage was to move the manipulator close to the object. The remote controller gave out end-effector velocity commands. The robot generated the movements adaptively using algorithms presented in Motion Distribution Algorithm Using Task Prioritization. The second stage was to set the base in balance stand and use the manipulator to pick up the object. The third stage was to move to the next target place. During this stage, the robot used the locomotion ability of its quadruped base. Fig. 10 shows the snapshots of the experiment. It shows that the robot can successfully pick up the object while maintaining its stableness. Fig. 11 shows the tracking errors during the first stage with the maximum value of 2.9 cm in the *x* axis and 3.2 cm in the *y* axis. The experiment shows that with the proposed framework, the robot can automatically coordinate its motion to stably finish object grasping and locomotion.

## Conclusion

A coordinated motion distribution and control framework is proposed in this paper. It can automatically distribute its end-effector motion to the base and the manipulator while maintaining stable locomotion. The quadruped base will be activated on the condition that the manipulator cannot finish the desired tasks or comes close to a singularity. The framework uses 3 prioritized tasks to distribute as many as possible motions to the manipulator, namely, end-effector trajectory tracking, adaptive motion distribution, and singularity avoidance. The null-space projection method is used in both motion generation and execution. The effectiveness of the framework is validated by several experiments on our robot. The robot can finish trajectory tracking both in and out of the workspace of the manipulator with good accuracy.

In the near future, we will extend our framework to plan the manipulation trajectory online. Vision-based recognition will be added to automatically finish grasping of objects. Furthermore, we want to extend this work for confined space manipulation and highly dynamic object catching through reinforcement learning.

## Data Availability

The data that support the findings of this study are available from the corresponding author upon reasonable request.

## Supplementary Materials

Supplementary 1 Videos of the experiments are available as Movie 1: https://youtu.be/qRY_pgJ6JJQ.
